# Multivariate modeling of settling depth of apple fruit (Red Delicious variety) in water

**DOI:** 10.1002/fsn3.265

**Published:** 2015-08-18

**Authors:** Kamran Kheiralipour, Farshid Marzbani

**Affiliations:** ^1^ Mechanical Engineering of Biosystems DepartmentIlam UniversityIlamIran

**Keywords:** Hydraulic handling, multivariate modeling, physical characteristics, settling depth

## Abstract

Settling depth of targets, with a density lower than the density of water, is a distance between water surface and a depth that the target reaches after dropping from a height. In this research, settling depth of apple was determined by a water column and a digital camera and then was experimentally modeled using multivariate regression program coded in MATLAB software. The considered parameters in multivariate modeling were physical characteristics (density, mass, and volume) and dropping height of the target. The characteristics were determined by standard methods. The best models were based on the density, dropping height and volume/mass with coefficient of determination (*R* ^2^), and mean square error (MSE) of 0.90 and 4.08, respectively.

## Abstract 2

Settling depth of apple was determined by a water column and a digital camera. The depth was experimentally modeled using multivariate regression using a coded program in MATLAB software. The best models were based on the density, dropping height volume/mass with coefficient of determination and mean square error of 0.90 and 4.08, respectively.

## Introduction

Engineering properties of agricultural materials are important information required to design handling, cleaning, conveying, grading, packaging, and storage systems (Tabatabaeefar 2003). Settling depth of fruits and vegetables in water is of the hydrodynamic properties defined for targets with density lower than the density of water. It is a depth that a target dropped from a height will reaches to that and returns to the water surface. Settling depth of fruits and vegetables is necessary for determining the depth of water in channel to avoid the fruit contact with channel bottom (Mohsenin 1986).

Kheiralipour (2014) theoretically modeled the settling depth of fruits and vegetables with the density lower than the density of water, *ρ*_*f*_ **< *ρ*_*w*_, as following formula:

(1) $$d=\frac{2d_{0}\left(\rho_{f}-\rho_{a}\right)V^{\frac{n+1}{3}}}{\left(\rho_{w}-\rho_{f}\right)V^{\frac{n+1}{3}}+K\mu_{w}^{n}\rho_{w}^{1-n}S_{h}{\left(2gd_{0}\left(1-\frac{\rho_{a}}{\rho_{f}}\right)\right)}^{\frac{2-n}{2}}}$$

where *d* is settling depth of fruit, *d*_*0*_ is dropping height, *ρ*_*f*_ is target density, *ρ*_*a*_ is air density, *V* is target volume, *ρ*_*w*_ is water density, *μ*_*w*_ is the static viscosity of water, *S*_*h*_ is shape factor, *g* is acceleration of gravity, and *k* and *n* are constant factors. The more important effective parameters on settling depth are dropping height, density, mass, and volume of target (Kheiralipour 2014).

In the world, the total production of apple fruit (*Malus domestica* Borkh L.) is around 76.38 million tons. China is the first apple producer in the world with 37 million tons annual production. After United States, Turkey, Poland, India, and Italy, Islamic Republic of Iran with production of 17.00 million tons is ranked as the seventh producer country (FAOSTAT, 2012). In the literature, physical characteristics of apple fruit were investigated and reported (Tabatabaeefar and Rajabipour 2005; Kheiralipour et al. 2008; Jalali et al. 2013) but there is no information about settling depth of apple fruit. So, the decision in this research was to develop experimental model of apple settling depth based on its physical properties. The settling depth of apple, Red Delicious variety, was determined and modeled considering theoretical model developed by Kheiralipour (2014) and multivariate regression procedure.

## Materials and Methods

### Theory

In this research, equation (1) was considered for experimental modeling of settling of targets in water. By reversing *d,* equation (1) is changes to:

(2) $$\frac{1}{d}=\frac{\left(\rho_{w}-\rho_{f}\right)V^{\frac{n+1pt1}{3}}+K\mu_{w}^{n}\rho_{w}^{1-n}S_{h}{\left(2gd_{0}\left(1-\frac{\rho_{a}}{\rho_{f}}\right)\right)}^{\frac{21pt-1ptn}{2}}}{2d_{0}\left(\rho_{f}-\rho_{a}\right)V^{\frac{n1pt+1pt1}{3}}}$$

Equation (2) can be broken as following:

(3) $$\frac{1}{d}2pt=2pt\frac{\left(\rho_{w}-\rho_{f}\right)V^{\frac{n1pt+1pt1}{3}}}{2d_{0}\left(\rho_{f}-\rho_{a}\right)V^{\frac{n1pt+1}{3}}}+\frac{K\mu_{w}^{n}\rho_{w}^{11pt-1ptn}S_{h}{\left(2gd_{0}\left(1-\frac{\rho_{a}}{\rho_{f}}\right)\right)}^{\frac{21pt-1ptn}{2}}}{2d_{0}\left(\rho_{f}-\rho_{a}\right)V^{\frac{n1pt+1pt1}{3}}}$$

After simplification, equation (3) would be changed as following:

(4) $$\frac{1}{d}1.5pt=1.5pt\frac{1}{2d_{0}}\frac{\left(\rho_{w}-\rho_{f}\right)}{\left(\rho_{f}-\rho_{a}\right)}+\frac{K\mu_{w}^{n}\rho_{w}^{11pt-1ptn}S_{h}{\left(2gd_{0}\right)}^{\frac{21pt-1ptn}{2}}}{2d_{0}}\frac{{\left(\rho_{f}-\rho_{a}\right)}^{-n}V^{-\frac{n1pt+1pt1}{3}}}{\rho_{f}}$$

The parameters $\frac{1}{2d_{0}}$ and $\frac{K\mu_{w}^{n}\rho_{w}^{11pt-1ptn}{\left(2g\right)}^{\frac{21pt-1ptn}{2}}}{2}$ in equation (4) are replaced by *K*_2_ and *K*_3_. So equation (5) can be:

(5) $$\frac{1}{d}2pt=2ptK_{2}{d_{0}}^{-1}\frac{\left(\rho_{w}-\rho_{f}\right)}{\left(\rho_{f}-\rho_{a}\right)}+K_{3}S_{h}{d_{0}}^{-n}\frac{{\left(\rho_{f}-\rho_{a}\right)}^{-n}V^{-\frac{n1pt+1pt1}{3}}}{\rho_{f}}$$

The air density (*ρ*_*a*_) can be neglected in previous equation and *K*_2_ *ρ*_*w*_ = *K*_1_, so:

(6) $$\frac{1}{d}2pt=2ptK_{1}{d_{0}}^{-1}{\rho_{f}}^{-1}-K_{2}{d_{0}}^{-1}+K_{3}S_{h}{d_{0}}^{-n}{\rho_{f}}^{-1pt1-1ptn}V^{-\frac{n1pt+1pt1}{3}}$$

The volume in the equation (6) can be replaced by target mass, *m*, as following:

(7) $$\frac{1}{d}2pt=2ptK_{1}{d_{0}}^{-1}{\rho_{f}}^{-1}-K_{2}{d_{0}}^{-1}+K_{3}S_{h}{d_{0}}^{-n}{\rho_{f}}^{\frac{-2n1pt-1pt2}{3}}m^{-\frac{n1pt+1pt1}{3}}$$

So, descent depth of target can be modeled using equations (8) or (9):

(8) $$\frac{1}{d}2pt=2ptA{d_{0}}^{-1}{\rho_{f}}^{-1}+BS_{h}{d_{0}}^{C}{\rho_{f}}^{D}V^{E}+F{d_{0}}^{-1}+G$$

(9) $$\frac{1}{d}2pt=2ptA{d_{0}}^{-1}{\rho_{f}}^{-1}+BS_{h}{d_{0}}^{C}{\rho_{f}}^{D}m^{E}+F{d_{0}}^{-1}+G$$

where *A*,* B*,* C*,* D*,* E*,* F,* and *G* are constant factors. The constant coefficient *G* was added to the end of the equations (8) and (9) as an error in experimental modeling. The difference between equations (8) and (9) is volume and mass, only.

## Experiments

The targets in this research were apple fruit from Red Delicious variety. Target mass was determined by Precisa electronic balance (Model: 3100c) with accuracy of 0.1 g. Volume and density of the targets were determined by the water displacement method (Mohsenin 1986).

**Figure 1:**
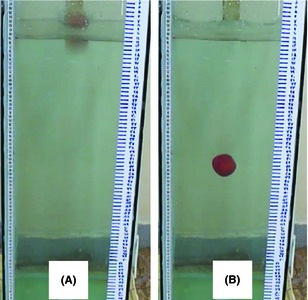
Traveling a target from (A) water surface to (B) its settling depth.

A water column (35 × 35 × 90 cm^3^) was constructed by glass with thickness of 8 mm. It was filled by water to a height of 80 cm. Each target was placed on a specific height (dropping height = 10, 25, and 50 cm) on top of the water surface, so that the largest areas of them were parallel to the surface of the water column. In order to determine settling depth of the targets a digital camera (Sony, Model) with 25 frames per second recorded the moving of them from the dropping height point to the end of the target's settling depth in water column. Each fruit was tested three times in each dropping height. In order to correctly determine the settling depth of the fruits, Video to Frame Software was used to change each video film to corresponding images (Fig. 1).

### Multivariate modeling

The obtained data were considered for modeling of settling depth of apples using equation (8) and (9). In the present research, the projected area of the targets was not determined due to low effect on the terminal velocity (Jordan and Clark 2004; Kheiralipour et al. 2010). So, the equations (8) and (9) can be changed to:

(10) $$\frac{1}{d}2pt=2ptA{d_{0}}^{-1}{\rho_{f}}^{-1}+H{d_{0}}^{C}{\rho_{f}}^{D}V^{E}+F{d_{0}}^{-1}+G$$

(11) $$\frac{1}{d}2pt=2ptA{d_{0}}^{-1}{\rho_{f}}^{-1}+H{d_{0}}^{C}{\rho_{f}}^{D}m^{E}+F{d_{0}}^{-1}+G$$

where, *H* is a new constant factor instead of *BS*_*h*_.

The multivariate regression method was used for modeling settling depth of the targets. For this, a program was coded in MATLAB Software. The program was applied “nlinfit” command in the software. The program was able to calculate determination coefficient (*R* ^2^) and mean square error (MSE) of each model.

## Results and Discussions

Some physical properties of the targets were presented in Table 1. In this table, besides density, volume and mass, settling depth of the targets when dropping from the different heights of 10 cm (*d*_10_), 25 cm (*d*_25_), and 50 cm (*d*_50_) were listed.

**Table 1:** Some physical properties of the targets

	Minimum	Mean	Maximum	Standard deviation
Density (g/cm^3^)	0.82	0.86	0.88	0.02
Volume (cm^3^)	123.78	162.64	211.00	25.84
Mass (g)	104.44	139.40	171.94	20.32
*d*_10_ (cm)^a^	25.7	30.3	34.0	2.2
*d*_25_ (cm)^a^	33.9	38.0	42.0	2.0
*d*_50_ (cm)^a^	39.0	44.6	51.0	3.4

a *d*_10_, *d*_20_ and *d*_50_ are settling depth of the targets when dropping from a height of *d*_0_ = 10, 25 and 50 cm, respectively.

**Figure 2:**
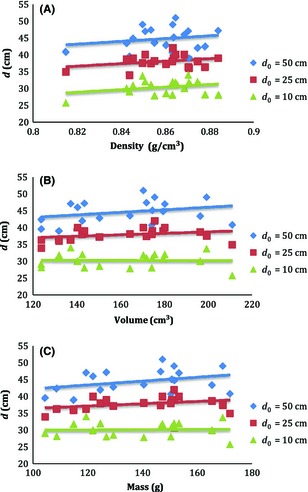
The settling depth of targets versus (A) fruit density, (B) volume, and (C) fruit mass. *d* is settling depth and *d*_*0*_ is dropping height.

Settling depth of all experimented targets was plotted in Figure 2. As shown in this figure, by increasing the dropping height from 10–50 cm, the settling depth was increased. As can be seen in this figure, the settling depth of all targets was not uniformly increased. This is due to effect of other parameters of target on the settling depth.

As can be seen in this figure, most of the lines to show the relations between settling depth and physical attributes of the fruit have increasing trend by the increase in target mass, volume, and density. This trend is more detectable for Figure 2A. Also by comparing all parts of Figure 2, it is observed that the effect of dropping height is higher than that of mass, volume, and density of the target.

Multivariate models of settling depth of the targets were done based on equations (10) and (11) for each dropping height individually. The models were shown in Tables 2, 3, 4 for dropping height of 10, 25, and 50 cm, respectively.

**Table 2:** Multivariate models of the settling depth of the targets with dropping height of 10 cm

Model No.	Model	MSE	*R* ^2^
1	$\frac{1}{d}=0.0362{\rho_{f}}^{-1}-0.0090$	5.2246 × 10^−6^	0.1021
2	$\frac{1}{d}=6.9800\;\;\times \;10^{-15}V^{4.87}+0.033$	5.7015 × 10^−6^	0.0199
3	$\frac{1}{d}=8.2200\;\;\times \;10^{9}m^{-6.43}+0.033$	5.7650 × 10^−6^	0.0090
4	$\frac{1}{d}=341.2355{\rho_{f}}^{-163.8828}V^{-8.3104}+0.0323$	3.8646 × 10^−6^	0.3358
5	$\frac{1}{d}=344.6594{\rho_{f}}^{-155.6381}m^{-8.3149}+0.0328$	3.8645 × 10^‐6^	0.3358
6	$\frac{1}{d}=-0.2955{\rho_{f}}^{-1}+0.0067{\rho_{f}}^{-13.4032}V^{-0.1310}+0.03502$	3.5561 × 10^−6^	0.3994
7	$\frac{1}{d}=-0.2955{\rho_{f}}^{-1}+0.0067{\rho_{f}}^{-13.4032}m^{-0.1310}+0.3502$	3.5561 × 10^−6^	0.3994

**Table 3:** Multivariate models of the settling depth of apple with dropping height of 25 cm

Model No	Model	MSE	*R* ^2^
1	$\frac{1}{d}=0.0205{\rho_{f}}^{-1}+0.0025$	1.6152 × 10^−6^	0.1057
2	$\frac{1}{d}=2.9694\times 10^{10}V^{-6.2481}+0.0257$	1.2514 × 10^−6^	0.3068
3	$\frac{1}{d}=1.7067\times 10^{10}m^{-6.3113}+0.0256$	1.1576 × 10^−6^	0.3588
4	$\frac{1}{d}=0.2515{\rho_{f}}^{-0.3194}V^{-0.0373}-0.1920$	6.7594 × 10^−6^	0.6256
5	$\frac{1}{d}=0.2020{\rho_{f}}^{-0.3658}m^{-0.0484}-0.1419$	6.8423 × 10^−6^	0.6256
6	$\frac{1}{d}=0.0631{\rho_{f}}^{-1}+2.2925\times 10^{4}{\rho_{f}}^{6.7748}V^{-2.9721}-0.0498$	5.5090 × 10^−6^	0.6948
7	$\frac{1}{d}=0.1172{\rho_{f}}^{-1}+0.7941{\rho_{f}}^{6.0461}m^{-0.6364}-0.1228$	5.9653 × 10^−6^	0.6699

**Table 4:** Multivariate models of the settling depth of the targets with dropping height of 50 cm

Model No	Model	MSE	*R* ^2^
1	$\frac{1}{d}=0.0159{\rho_{f}}^{-1}+0.0040$	2.7568 × 10^−6^	0.0398
2	$\frac{1}{d}=1.0505\times 10^{10}V^{6.0249}+0.0218$	2.2829 × 10^−6^	0.2048
3	$\frac{1}{d}=1.3282\times 10^{10}m^{-6.2536}+0.0217$	2.2035 × 10^−6^	0.2324
4	$\frac{1}{d}=1.0841{\rho_{f}}^{-0.0631}V^{-0.0081}-1.0299$	1.7423 × 10^−6^	0.3942
5	$\frac{1}{d}=1.7559{\rho_{f}}^{-0.0385}m^{-0.0049}-1.6999$	1.7409 × 10^−6^	0.3942
6	$\frac{1}{d}=0.0977{\rho_{f}}^{-1}+1.4534{\rho_{f}}^{5.6407}V^{-0.8112}-0.1015$	1.6627 × 10^−6^	0.4211
7	$\frac{1}{d}=0.0977{\rho_{f}}^{-1}+1.4536{\rho_{f}}^{5.6409}m^{-0.8112}-0.1015$	1.6625 × 10^−6^	0.4211

In Table 2, model No. 1, 2, and 3 (single‐variate) with *R* ^2^ = 0.1021, 0.0199, and 0.0090, respectively, show that density, volume, and mass of the targets cannot individually model the settling depth from 10 cm dropping height. Also other models which are multivariate, with a coefficient determination of 0.3358–0.3994 cannot strongly predict the settling depth with 10 cm dropping height.

Models No. 1, 2, and 3 in Table 3, are same as those in Table 2. The other models in Table 3 have the determination coefficients higher than the corresponding values of Table 2.

Also, Models No. 1, 2, and 3 in Table 4 have low determination coefficient same as corresponding values in Tables 2 and 3. The other models show that settling depth of apple dropped from height of 25 cm can be better modeled than the settling depth of targets dropped from the height of 10 cm (Table 2).

Settling depth of the targets was modeled based on equations (10) and (11) but with considering all dropping heights (Table 5).

**Table 5:** Final multivariate models of settling depth of apple

Model No	Model	MSE	*R* ^2^
1	$\frac{1}{d}=-234.4133{d_{0}}^{-1}{\rho_{f}}^{-1}+0.0019{d_{0}}^{0.7568}{\rho_{f}}^{10.9868}V^{1.4870}-209.6996{d_{0}}^{-1}+32.7118$	4.0752	0.9004
2	$\frac{1}{d}=-234.4135{d_{0}}^{-1}{\rho_{f}}^{-1}+0.0019{d_{0}}^{0.7568}{\rho_{f}}^{9.4998}m^{1.4870}-209.6996{d_{0}}^{-1}+32.7118$	4.0752	0.9004

As can be seen in Table 5, the coefficient of determination and mean square error of the two models is same and equal to 0.9004 and 4.0752, respectively. By this result it can be told that both parameter groups ([1] dropping height, density and volume and [2] dropping height density and mass) can model the settling depth with same ability. Also, these models have higher coefficient of determination compare with the models in Tables 2, 3, 4.

The models in Table 5 can be used to predict the settling depth of apple, Red Delicious variety. For hydraulic handling and transporting of apple when dropped from a specific height, the settling depth can be predicted to avoid the fruit impact, for example, with channel bottom.

## Conclusions

Some physical characteristics, density, volume, and mass of apple variety were determined. The settling depth of the dropped target from different heights of 10, 25, and 50 cm was determined using a water column and a digital camera. The settling depth was experimentally modeled based on the determined parameters. Based on the best model, *R* ^2^ = 0.9004 and MSE = 4.0752.

## Conflict of Interest

None declared.
